# Danger zone analysis using cone beam computed tomography after apical enlargement with K3 and K3XF in a manikin model

**DOI:** 10.4317/jced.52523

**Published:** 2016-10-01

**Authors:** Juan-Gonzalo Olivier, Marc García-Font, Jose-Antonio Gonzalez-Sanchez, Miguel Roig-Cayon, Fernando Durán-Sindreu

**Affiliations:** 1 DDS, PhD. Department of Restorative Dentistry and Endodontics, Universitat Internacional de Catalunya; 2 MD, PhD. Department of Restorative Dentistry and Endodontics, Universitat Internacional de Catalunya

## Abstract

**Background:**

The objective of the study was to evaluate and compare how apical enlargement with K3 and K3XF nickel-titanium (NiTi) rotary instruments reduces the root thickness in the danger zone and affects canal transportation and centering ability in mandibular molar mesial canals in a manikin extracted tooth model.

**Material and Methods:**

Seventy-two mesial root canals of first mandibular molars were instrumented. Initial and post-instrumentation Cone Beam Computed Tomography scans were performed after root canal preparation up to size 25, 30, 35 and 40 files. Canal transportation, canal centering and remaining root dentin thickness toward the danger zone were calculated in sections 1, 2 and 3 mm under the furcation level. Data were analyzed using non-parametric Kruskal-Wallis analysis of variance at a significance level of *P* < 0.05.

**Results:**

K3 instruments removed more dentin toward the danger zone compared with K3XF instruments (*P*< .05) and significant differences in dentin thickness were found when canal enlargement was performed to a #35-40 with both systems (*P*< 0.05). No significant differences in canal transportation and centering ability were found between systems, except when canal enlargement was performed to a #40 (*P* = 0,0136). No differences were observed when comparing the number of uses in both systems (*P*> 0.05).

**Conclusions:**

Under the conditions of this study K3 removed a significant amount of dentin at the furcation level compared with the R-Phase K3XF rotary system in curved root canals. Enlargement to a 35-40/04 file removed significantly more dentin with both systems.

** Key words:**K3, K3XF, R-phase, center ability, canal transportation, dentin thickness, increased apical enlargement, danger zone, dentin thickness.

## Introduction

The mandibular first and second molars have a distal concavity in the mesial root. Located under the furcation level it was described as the danger zone by Abou-Rass and Glick (1). This concavity is closer to the mesial canals than what can be determined by buccolingual radiographs (1,2). Mean distance from the wall of mesial root canals to the distal surface of the root ranges from 0.7 to 1.27 mm (2-5). Coronal flaring removes interferences and allows better control of the instruments in the one-third of the root canal (6). Additionally, coronal flaring provides better penetration of the irrigation needle, improving the efficiency of the irrigating solutions (6,7). However, care must be taken to avoid excessive dentin removal with overflaring (1). Root thickness tends to decrease considerably in this area during canal shaping and it is particularly prone to excessive weakness and iatrogenic damage including strip perforation (3).

According to the Glossary of Terms of the American Association of Endodontists, strip perforation is the complete penetration of a root canal wall due to excessive lateral tooth structure removal during canal preparation. However, even when strip perforation does not occur, a reduced thickness after instrumentation may lead to perforation or fracture during obturation (8). Furthermore, the strength and ability of the tooth to resist lateral forces to avoid root fracture are directly related to the remaining root thickness (9). Thus, anticurvature debridement of the root canal should be performed toward the mesial surface of mandibular molar mesial roots (1). Typically, less-tapered instruments are used for apical enlargement so that the instrument does not engage in the coronal and middle thirds and can perform safely within the WL. However, even with instruments that are less tapered, canal curvature may lead to a continuation of the removal of dentin in the danger zone when apical enlargement is carried out.

Instruments with higher bending values produce more centered preparations (10,11). However, few studies have evaluated root canal shaping behavior of instruments with the same geometrical characteristics that differ only in the alloy itself (12,13). K3 (Sybron Endo; Glendora, CA) and K3XF (Sybron Endo) instruments have the same geometric characteristics; constant taper design with an asymmetrical 3-fluted cross-section with unequally spaced flutes and recessive surfaces. These two instruments differ only in the post-matching heat treatment. The R-Phase Technology (SybronEndo; Glendora, CA) has achieved increased angular deflection to failure (10), superelasticity and cyclic fatigue resistance in both reciprocating (14) and rotational motion (10,15) of K3XF (SybronEndo) instruments compared with K3 (SybronEndo). Both instruments have the same geometrical characteristics (a U-shaped cross section with three cutting blades, three flutes with sinuous profiles and three radial lands) (10).

Several methods have been used to evaluate root canal shaping by endodontic instruments. These methods include cross-sectioning (16), radiographic imaging (17), and computed tomography methods (18-20). Cone Beam Computed Tomography (CBCT) scanning permits evaluation of canal preparations before, during and after canal shaping, providing high-resolution images and especially in the coronal and middle thirds of the root and not as much in the apical third. Changes in structures and root thickness can be evaluated without the destruction of specimens (19).

The purpose of this study was to evaluate and compare how apical enlargement with K3 and K3XF nickel-titanium (NiTi) rotary instruments reduces the root thickness in the danger zone and affects canal transportation and centering ability in mandibular molar mesial canals in a manikin extracted tooth model.

## Material and Methods

The present research protocol was approved by the Research Ethics Committee of the Universitat Internacional de Catalunya, Barcelona, Spain.

-Specimen preparation

First mandibular human molars with two curved mesial root canals with separate foramina were selected. Samples were stored immersed in saline solution. The access cavities were prepared and the distal roots of all teeth were removed with a stainless-steel disc.

A 10 K-file size was passively placed in the canal until the tip of the instrument was visibly adjusted to the apical foramen under the microscope. The WL was determined by subtracting 1 mm from this measurement. The teeth were then shortened and the rubber stop was adjusted to a flat anatomic landmark standardizing the WL measurement at 19 mm for all teeth. Preoperative X-rays with a 15 K-file to the WL were performed. AutoCAD 2011 (Autodesk Inc., San Rafael, CA) was used to determine the angle and radius of curvature of each root canal according to the methodology of Pruett *et al.* (21). Only root canals with a radius of curvature ranging between 4 mm and 9 mm and whose angles of curvature ranged between 20° and 35° were included.

A customized silicone jig was designed to ensure a constant position during pre- and post-instrumentation CBCT scanning. Initial CBCT scans were performed (Promax 3D; Planmeca, Helsinki, Finland) with constant exposure parameters of 90 kV, 12.0 mA and 12.23 sec. A 4 x 4 cm field of view was selected with a pixel size of 100 µm. Samples with mesial canals with isthmus were discarded.

A total of seventy-two root canals were included in the study. The root canals were allocated into two identical groups of 36 root canals (18 MB and 18 ML) according to dentin thickness to the risk zone and the degree and the radius of curvature.

Each tooth was placed in the first mandibular molar position of a typodont placed in an adult dental manikin as described in a previous study (13) and ensuring access to the root canal could only be gained from the same direction. All the instrumentation procedure was performed by an operator trained in both systems. All instruments used in the study were autoclaved before use.

-Canal preparation

A manual glide path up to a #20 K-file was performed before mechanical instrumentation. Root canals in group A were prepared with K3XF instruments and root canals in group B with K3 instruments. Subgroups A1-A2 and B1-B2 were created according to the first and second instrument use. Each instrument was used to shape 1 MB and 1 ML canals. Root canals were prepared in a crown-down technique with a 16:1 reduction handpiece powered by a torque-limited electric motor (Endo-Mate DT, NSK Europe, Frankfurt, Germany) as follows: 25.08: 14 mm; 25.06: 17 mm; and 25.04, 30.04, 35.04 and 40.04 files to the WL (19 mm). After every file that reached the WL a post-instrumentation CBCT scan was performed.

Canals were irrigated with 2 mL of 4.2% NaOCl using a 30-gauge Max-i-Probe (Dentsply, Tulsa, USA) after each file during instrumentation and were kept flooded during instrumentation. Patency was verified after every step with a #10 K-file.

-Sample analysis

**Figure 1:**
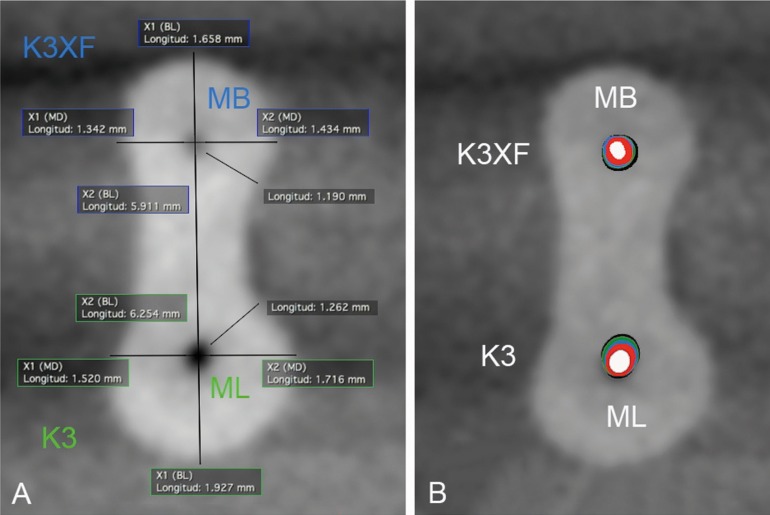
A) An example of measurements obtained from the CBCT images for measurement of canal transportation, centering ability and dentin removal toward the danger zone. B) An example of a CBCT section showing the original canal in white and the prepared outline in red (25.04), blue (30.04), green (35.04), black (40.04). MB canal prepared with K3XF instruments and ML canal with K3 instruments.

The section images of 1, 2 and 3 mm under the furcation level were evaluated. The distances between the root canal to the mesial, distal, buccal and lingual borders were measured in a 600% zoom (Fig. 1). For each section, the canal transportation (x1–x2)–(y1–y2) and the centering ratio (x1–x2)/(y1–y2) or (y1–y2)/(x1–x2) were calculated for the mesiodistal and the buccolingual displacement using the formula ratio proposed by Gambill *et al.* (22). The shortest distance from the edge of the root to the edge of the uninstrumented canal were represented by x1 and x2 (Fig. 1) and post instrumentation measurements by y1 and y2. In addition, dentin thickness toward the danger zone was calculated by measuring the minor distance from the edge of the root canal to the external surface of the root distal concavity (8).

-Statistical analysis

Values of central tendency and dispersion were calculated using Statgraphics Centurion XV software (Statpoint Technologies, Warrenton, USA) and data were analyzed using non-parametric Kruskal-Wallis analysis of variance at a significance level of *P* < 0.05.

## Results

Statgraphics Centurion XV software (Statpoint Technologies, Warrenton, USA) was used for all statistical analysis. Analysis of variance revealed no significant difference within groups regarding the angle, radius of curvature and the dentin thickness to the danger zone before canal preparation (*P* > .05).

-Canal Transportation

At the 2 mm level under the furcation, K3XF canal shaping resulted in a statistically significant lower canal transportation compared with K3 and in the mesiodistal direction when instrumentation was performed up to a size 40.04 (*P* = 0,0136). No statistically significant differences were found at the other studied levels. According to the number of uses no statistically significant differences were found between groups (*P* > 0.05) ( Table 1).

**Table 1:**
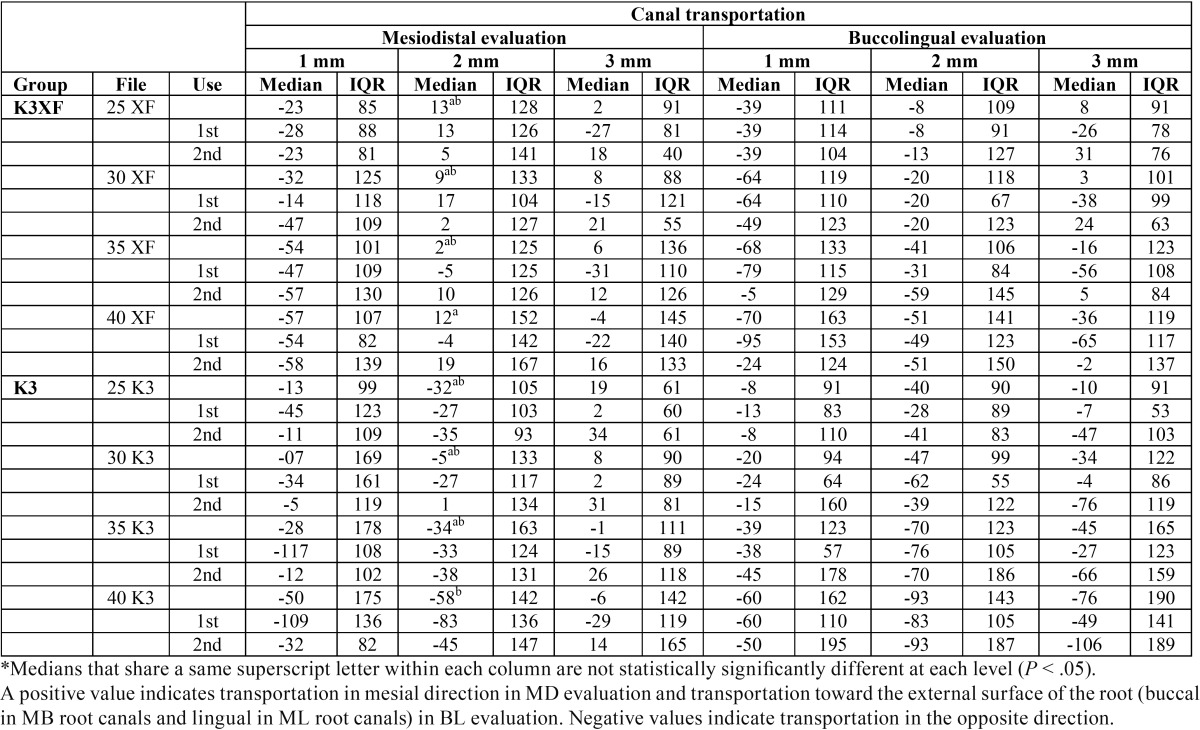
Comparison of canal transportation 1, 2 and 3 mm under the furcation level in the mesiodistal (MD) and buccolingual (BL) directions. Median and Interquartile range (IQR) in µm.

-Centering Ratio

At the 1 and 3-mm levels under the furcation, there was no statistically significant difference in the centering ratio among the groups (*P* > .05). However, at the 2-mm level, in the K3XF group a significantly higher mean centering ratio was recorded when instrumentation was performed up to a size 40.04 compared with a size 25.04 in both MD*P* = 0,0025) and BL directions (*P* = 0,0007). No statistically significant differences were found according to the number of uses (*P* > 0.05) ( Table 2).

**Table 2:**
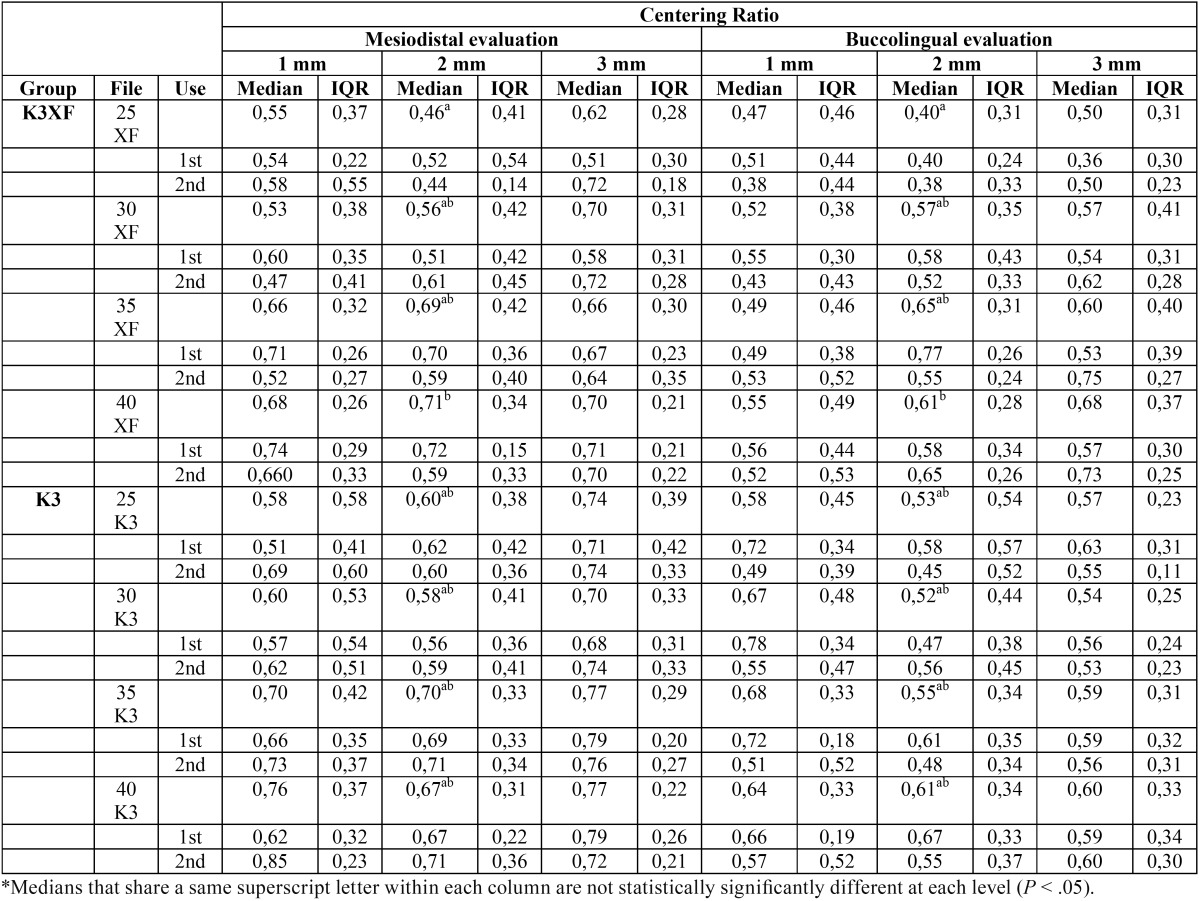
Statistical analysis of Centering Ratio values for tested groups 1, 2 and 3 mm under the furcation level in the mesiodistal (MD) and buccolingual (BL) directions.

-Removed Dentin toward the Danger Zone

At the three levels evaluated K3 removed more amount of dentin toward the danger zone. However differences were only statistically significant between systems in the 2-mm level under the furcation when instrumentation was performed up to a size 40.04 (*P* = 0,0039). According to the instrument size, in both systems differences were found when instrumentation was performed up to a size 35.04 and 40.04 compared with 25.04 and 30.04 (*P* < 0.05) ( Table 3).

**Table 3:**
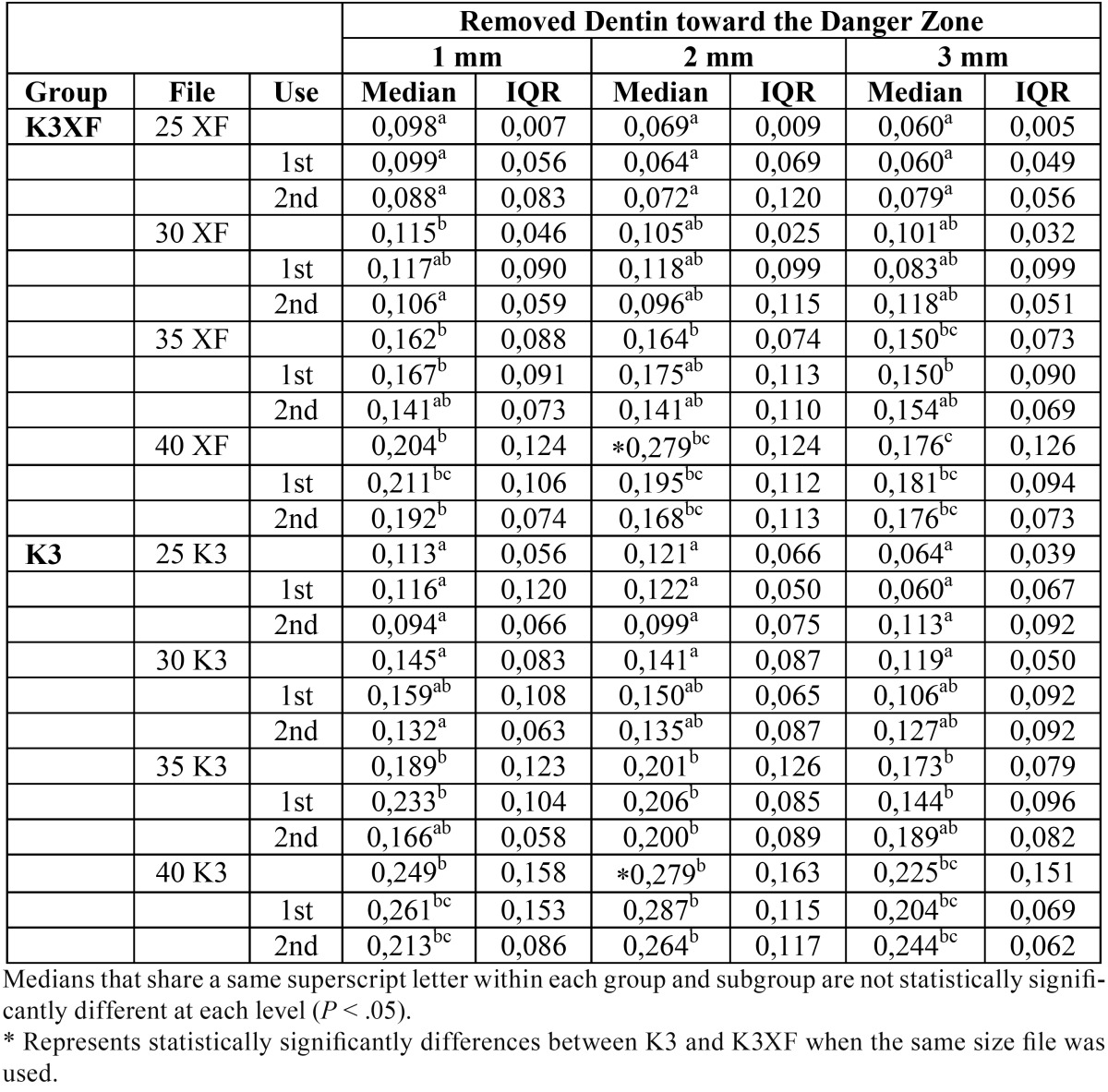
Comparison of Removed Dentin toward the Danger Zone for tested groups 1, 2 and 3 mm under the furcation. Median and interquartile range (IQR) in mm.

## Discussion

Crown-down techniques have been proposed when shaping root canals. Preflaring before reaching the WL permits apical enlargement with less transportation and procedural errors (3,6). However, an excessive coronal shaping may lead to iatrogenic complications, such as perforations and stripping, particularly in the inner surface of the curve (1,3). The excessive structure loss may also lead to a reduced resistance to root fracture under functional loads (9).

Although no strip perforations occurred during the study, little remaining tooth structure in some cases could be observed. However, none of the residual root thicknesses for any of the surfaces and sections in this study resulted less than 0.54 mm. Lim & Stock (23) recommended that the remaining dentine thickness should be no less than 0.3 mm to withstand forces during canal filling.

Variations in canal anatomy have more influence on the post-operative canal geometry than the rotary system used for canal shaping (24). Despite the morphologic variations of natural teeth, effort was made to ensure comparability between the experimental groups. Only mandibular first molars with similar root structures and two independent mesial canals were selected. In addition, the WL was standardized at 19 mm and the canal group was evenly distributed with respect to the dentin thickness and the angle and radii of curvature of the root canals before canal shaping.

Access to the root canal, especially in posterior teeth, may affect root canal shaping. In several studies teeth have been embedded in resin blocks (11,18,19). Thus, the root anatomy remains hindered like as in the bone socket. However, teeth position during canal preparation or anatomic structures that may alter instrumentation performance have not been taken into account. Thus, a more approximate methodology to clinical practice such as a manikin model should be used when evaluating instrumentation techniques (13,25). The attempt to replicate the clinical conditions, to assess the performance of shaping instruments in extracted teeth, should be the primary goal when designing an *in vitro* study (25). In addition, crowns were maintained for root canal preparation. Although decoronation has been performed in some *in vitro* studies to evaluate canal shaping, it has been stated that crown removal changes tension and instrument behavior (26). Additionally, it could interfere with the straight-line access to the canal, thereby influencing the cervical pre-enlargement (27).

According to Lim *et al.* (23) sections more than 3 mm under the furcation level were not evaluated. Under the conditions of their study, a greater risk of perforation into the furcation was found at a level 8 mm from the WL than at 5 mm. Moreover, the risk zone has been reported to be located between 4 and 6 mm below the canal chamber orifice (3), and Berutti and Fendon (5) found that the minimum dentin thickness was located between 1 and 2 mm under the furcation. The results in our study confirm these findings. The major dentin removal was produced in the 2-mm level under the furcation, highlighting the importance of performing safely at this level when shaping root canals to avoid risk of iatrogenic damage such as a strip perforation.

Increasing the root canal apical diameter allows a greater reduction in remaining bacteria and dentin debris when compared with smaller preparations (28,29). However it must be taken into account that as we increase apical preparations, the risk of stripping due to dentin loss also increases, and some authors advocate for small apical preparations (30,31). Thus, we measured dentin remaining thickness after instrumentation after every file that reached the WL. Although apical enlargement in this study was performed with small tapered instruments, root thickness under furcation was reduced significantly when instrumentation was performed up to a 40.04 file with both systems.

According to Gambill *et al.* (22), the mean centering ratio indicates the ability of the instrument to stay centered in the canal. The results from our study are consistent with other studies were rotatory NiTi instruments allow the preparation of curved canals with minimal risk of canal transportation and high centering ratios in the coronal one-third (19,20). Although canal shaping was per-formed in an anticurvature method, more dentine was removed from the inner curve surface. Bergmans *et al.* (32) concluded that this could be due to the bulk of cervical dentine that might force instruments toward the furcation.

K3XF instruments produced lower mean canal transportation. However results were only statistically significant in the mesiodistal direction when instrumentation was performed up to a 40/04 compared with K3 (*P* < .05). As the instruments used in this study are geometrically identical, differences can be attributed to the new manufacturing method, the R-phase. This technology increases instrument flexibility (15). Instrument flexibility has a determining influence on root canal shaping (33). A more flexible instrument might be able to prepare the apical one-third of the root canal enduring the tendency of the file to recover its initial lineal position.

Canal transportation and centering ability values do not display differences in dentin removal. As instruments remove dentin in all directions, an excessive amount removal could remain unnoticed if we only take into account formulas proposed by Gambill *et al.* (22). Thus we measured changes in dentin thickness towards the danger zone, were there is an increased risk of iatrogenic damage such as strip perforation (8).

Results of this study showed that K3 instruments removed more dentin toward the danger zone than K3XF instruments. This difference could explain the results from a previous study where K3 preparations required a statistically significant reduced preparation time compared with K3XF preparations with no differences in apical transportation (13). These results are consistent with Bergmans *et al.* (32). Under the conditions of their study, K3 instruments tend to remove a higher amount of dentin in the coronal third (0.48 mm3 ± 0.12) with minimal deviation in the apical one-third (4.14 µm ± 6.40). In addition, apical enlargement to a size 35.04 - 40.04 file significantly reduced more dentin with both systems (*P* < .05). Differences in instrument flexibility (15) may explain both the increase of dentin removal of K3 instruments near the furcation level compared with K3XF instruments and that enlarging apical preparations up to less flexible files may reduce considerably dentin thickness in the coronal one-third.

## Conclusions

Under the conditions of this extract tooth model, data analysis of the present study demonstrated the ability of rotary K3 and K3XF instruments to stay centered in the canal with minimal risk of transportation. However, the R-Phase K3XF Ni-Ti rotary files performed more safely near the danger zone in curved canals compared with the K3 instruments. Clinical advantages of the new manufacturing process, should allow for the preparation of curved canals with a reduced risk of canal transportation and iatrogenic errors.
